# Study on the Mechanical and Thermal Properties of Waterborne Polyurethane-Modified Aluminum Hydroxide and Its Application in LDPE Plastics

**DOI:** 10.3390/polym17050556

**Published:** 2025-02-20

**Authors:** Xianrong Yang, Gaoxiang Du, Huan Shuai, Xi Xu, Jiao Wang

**Affiliations:** 1 School of Materials Science and Technology, China University of Geosciences, Beijing 100083, China; 2103210011@email.cugb.edu.cn (X.Y.); shuaihuan@email.cugb.edu.cn (H.S.); 2103220017@email.cugb.edu.cn (X.X.); 2 Beijing Yiyi Star Technology Co., Ltd., Beijing 100089, China; 3 School of Basic Education, Beijing Polytechnic College, Beijing 100042, China; wj@bgy.edu.cn

**Keywords:** aluminum hydroxide, waterborne polyurethane, LDPE composites, mechanical properties, flame retardancy, modification

## Abstract

This study investigates the modification of aluminum hydroxide (ATH) powder using waterborne polyurethane (WPU) as a novel modifier, along with its subsequent effects on the dispersion, mechanical properties, and thermal performance of ATH-filled low-density polyethylene (LDPE) composites. ATH was modified through an optimized wet process, and the modification efficiency was evaluated using various characterization techniques, including scanning electron microscopy (SEM), X-ray diffraction (XRD), Fourier-transform infrared spectroscopy (FTIR), X-ray photoelectron spectroscopy (XPS), and thermogravimetric analysis (TGA). The results show that WPU, as a modifier, effectively improved the dispersion of ATH in the organic phase, as demonstrated by the reduced settling time and enhanced interfacial compatibility between ATH and LDPE. The modified ATH demonstrated enhanced mechanical properties in LDPE-based composites, with a tensile strength of 30.02 MPa, flexural strength of 13.20 MPa, impact strength of 65.75 kJ/m^2^, and elongation at break of 59.84%, all reaching their maximum at 3.0 wt.% WPU modification. Additionally, the flame retardancy of the composites was significantly improved due to the incorporation of ATH, with the ATH content in the composites reaching up to 60%, further enhancing their fire resistance. This study highlights the effectiveness of WPU-modified ATH as both a flame retardant and a reinforcing filler for LDPE composites, offering potential advantages in enhancing material properties while reducing manufacturing costs.

## 1. Introduction

Aluminum hydroxide (Al(OH)_3_, ATH) is a widely used, inorganic, halogen-free material known for its excellent flame-retardant properties, making it a popular filler in plastics [1,2,3,4] and coatings [3,5,6]. ATH makes polymer-based products more fire-resistant because of its endothermic decomposition process, which releases water vapor when it is heated. This absorbs a lot of heat, dilutes flammable gases, and helps prevent combustion, making ATH an effective flame retardant in polymer matrices. In plastics, ATH is commonly used in polymer systems like polypropylene (PP) [7] and low-density polyethylene (LDPE) [8].

Nonetheless, difficulties persist in the field of enhancing the dispersion and amalgamation of ATH with polymer matrices. This is primarily due to its hydrophilic character, which frequently results in suboptimal dispersion and aggregation within the polymer melt. Moreover, this often results in poor interfacial bonding between the filler and the polymer, leading to reduced mechanical performance and processing difficulties. While the addition of ATH may reduce tensile and impact strength, it enhances the bending strength of plastics, making it a versatile additive with both advantages and trade-offs in material formulations [9]. To address these issues, surface modification techniques have been explored to improve the dispersion of ATH and enhance its interaction with the polymer matrix. Surface modification can improve the compatibility of ATH with both organic and inorganic components, thus enhancing its efficiency as a flame retardant and improving the mechanical properties of the final composite materials.

In recent years, the surface modification of ATH has attracted growing global attention, leading to significant advancements. The phosphoric [1,2] and polyacrylic [10] acids, as well as silane [9], are commonly employed as modifiers to improve the interaction between ATH and polymer matrices. However, there is still a need to develop new modifiers that offer better performance, cost-efficiency, and environmental sustainability, which would further expand the functionality and industrial applications of ATH. Most studies have focused on using ATH as a filler in polymers at concentrations typically below 20 wt.% [8,9,11,12]. It is well known that modifying ATH for use as a filler often leads to a decline in the mechanical properties of the resulting polymer composites. Furthermore, in several studies, the ATH content has been kept relatively low, with some flame-retardant ATH additives reaching a maximum of only 15% [9], or even as low as 7% [4]. In terms of mechanical properties, composites with higher ATH content have shown reduced impact strength and flexural strength. This highlights the ongoing challenge of achieving higher ATH loadings without compromising the material’s mechanical integrity. Therefore, further research into the modification of ATH is essential to achieve composite materials with both stable mechanical properties and higher ATH incorporation. Successfully increasing the filler level would not only reduce polymer usage but also enhance the value-added utilization of ATH. Additionally, ATH is frequently used as a modifier in polyurethane foams [13], where it exhibits good compatibility. However, there has been limited research on ATH modified by polyurethane, indicating a promising avenue for future exploration.

In this study, the surface modification of ATH was applied using a waterborne polyurethane (WPU) prepolymer, aiming to investigate the mechanism and to improve its dispersion and compatibility in polymer systems, with a focus on enhancing the flame retardancy, mechanical performance, and cost-effectiveness of the resulting composites. The properties of ATH powder were assessed through tests, including contact angle and settling time. The effect of the modification on ATH’s affinity for the organic phase was assessed through ethanol dispersion, and the results were analyzed using scanning electron microscopy (SEM). The modification effect was further confirmed and analyzed using X-ray diffraction (XRD), Fourier-transform infrared spectroscopy (FTIR), thermogravimetric analysis (TGA), and X-ray photoelectron spectroscopy (XPS). The mechanical properties of the ATH-modified composites were tested, and the dispersion of ATH in the LDPE matrix was examined with SEM.

## 2. Experimental Section

### 2.1. Materials

**Figure 1:**
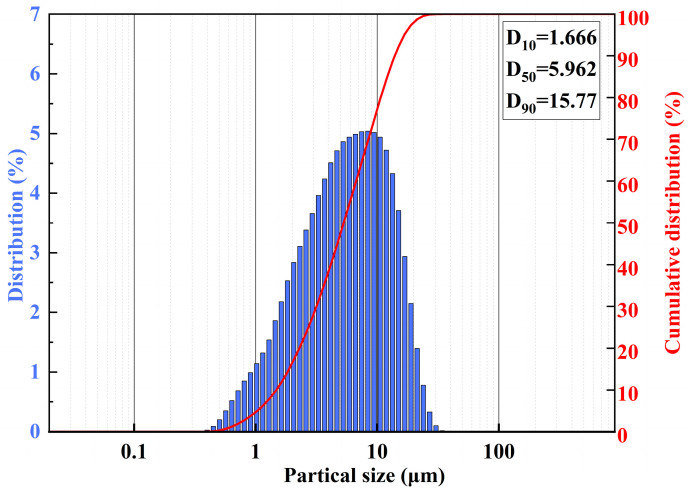
Particle size of ATH.

The material used was aluminum hydroxide (ATH) with a gibbsite crystal structure, composed of Al(OH)_3_, belonging to the monoclinic crystal system with an L2PC symmetry type. Its thermal decomposition begins at near 180 °C, and the particle size distribution is shown in Figure 1. It was supplied by Changge Tianlong Environmental Materials Co., Ltd. (Xuchang, China). The waterborne polyurethane prepolymer, a white emulsion formed through a prepolymerization [14,15] reaction between 1,4-butanediol and diisocyanate at a 1:1 molar ratio, had a solution density of 1.05 g/cm^3^, a viscosity of 100–300 mPa·s (at 23 °C), a pH range of 6–8, and a solids content of 30–32%. This polyurethane product was supplied by Huashi New Material Technology Co., Ltd. (Guangzhou, China). Low-density polyethylene (LDPE/LD165), supplied by Sinopec Yanshan Petrochemical Co., Ltd. (Beijing, China), has a density of 0.920–0.924 g/cm^3^, a melt index of 0.23–0.43 g/10 min, a melting point range of 106–111 °C, and a Vicat softening point above 92 °C. Kerosene was supplied by Jingtu Hengsheng Trading Co., Ltd. (Beijing, China), and distilled water (resistivity ≥ 18.2 MΩ·cm) was prepared in the laboratory.

### 2.2. Modification

The ATH particles were modified using a wet process. ATH powder and distilled water were mixed at a 1:4 weight ratio and placed in a beaker, followed by the addition of a specified amount (0.50–5.00%) of WPU to the mixture. The mixture was stirred at 1000 rpm for 15 min at room temperature, and then agitation was continued at 95 °C for 1 h. After that, the mixture was cooled to room temperature, filtered, dried at 90 °C, and packed for further use.

**Figure 2:**
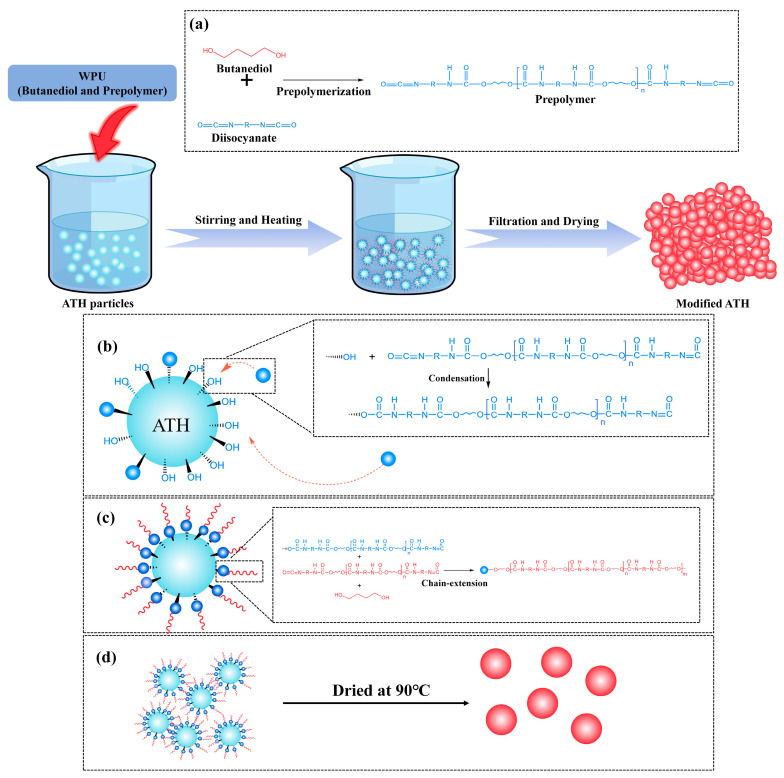
Mechanism of WPU-modified ATH: (**a**) The prepolymer is formed through the prepolymerization reaction between 1,4-butanediol and diisocyanate. (**b**) The prepolymer is grafted onto the surface of the ATH. (**c**) Butanediol and the prepolymer continue to condense to form polyurethane chains. (**d**) During the drying process, the polyurethane forms a coating structure on the surface of the ATH.

The proposed modification mechanism of WPU-modified ATH powder is shown in Figure 2. ATH powder contains a large number of hydroxyl groups on its surface. When stirred and mixed with WPU prepolymer, the mixture is heated to approximately 80 °C, at which point a condensation reaction occurs between the hydroxyl groups on the ATH surface and the isocyanate in the prepolymer [16,17,18,19]. After the prepolymer is grafted onto the ATH surface, continued condensation allows the remaining 1,4-butanediol to further react with the prepolymer on the ATH surface, thereby extending the polyurethane chains. The drying process promotes the crosslinking of the polyurethane chains on the ATH surface, forming a microcapsule-like coating structure to achieve the desired modification effect. The hydrophilicity of the modified ATH surface decreases due to the R-alkyl groups present in the diisocyanate, resulting in improved dispersion of ATH in the LDPE. This improvement in dispersion was also confirmed by the application of the modified ATH in LDPE plastics.

### 2.3. Preparation of LDPE-Based Composites

The composite materials were fabricated through a two-step process involving granulation and injection molding. Prior to this, particles of either modified or unmodified ATH and LDPE were meticulously mixed in precise proportions.

**Figure 3:**
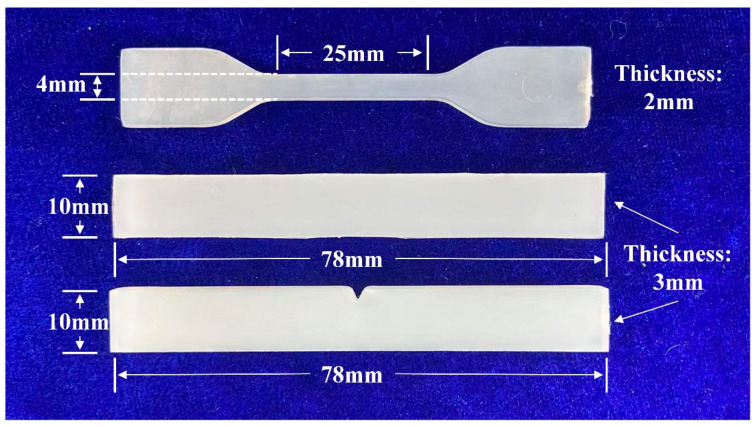
The dimensions of LDPE-based plastic composite products.

1. Mixing of raw materials: ATH and LDPE were added to a mixer according to the proportions shown in Table 1 and mixed at high speed for 10 min. The mixture was then packed for further use.

2. Granulation: A single-screw extruder and pelletizer were used for the granulation process. The mixed ATH and LDPE were extruded at 145 °C, and the molten mixture was cooled through a water bridge, cut into pellets, and packed for further use.

3. Injection molding: The pellets were melted at 145 °C and injection-molded to form specimens with the dimensions shown in Figure 3.

### 2.4. Characterizations

#### 2.4.1. Contact Angle

The measurement of the contact angle was conducted by employing a goniometer, utilizing the sessile drop technique as the method of measurement. The sample was pressed into a pellet, a 2 μL water droplet was placed on the surface, and the contact angle was calculated using the Young–Laplace method [20].

#### 2.4.2. Settling Time

This test evaluated the dispersion stability of ATH modified with different dosages of modifier in a non-polar environment, serving as an indicator of its dispersion performance in organic phases. A sample of powder (0.250 g ± 0.0005 g) was added to 50 mL of kerosene and stirred for 10 min using a magnetic stirrer. After stirring, 25 mL of the suspension was transferred to a measuring cylinder. The time taken for the 5.0 mL mark to become visible was recorded as the settling time.

#### 2.4.3. SEM Analysis

ATH powder (unmodified and modified with 3.0% WPU) was dispersed in ethanol at a concentration of 0.05 wt.% and subjected to ultrasonic treatment for 1 min. A 10 μL droplet of the dispersion was then placed on a silicon wafer, which was then gold-coated by sputtering at 20 mA for 120 s.

LDPE-based composites containing 20 wt.% ATH, both unmodified and modified with 3.0% WPU, were immersed in liquid nitrogen for 5 min. After removal, the samples were fractured in a brittle manner and then gold-coated under the same conditions (20 mA, 120 s).

The samples were examined using a Hitachi JSM-7610F field-emission scanning electron microscope, provided by JEOL Ltd. (Tokyo, Japan).

#### 2.4.4. XRD Analysis

X-ray diffraction (XRD) was performed to investigate the crystalline structure of ATH. Scanning was conducted at a speed of 10°/min over a 2θ range of 5–90°.

#### 2.4.5. FTIR Analysis

Fourier-transform infrared spectroscopy (FTIR) was carried out using a Bruker ALPHA FTIR spectrometer provided by the Bruker Corporation (Billerica, MA, USA). The spectral range for analysis was 4000–400 cm^−1^, which captured the key vibrational features of ATH before and after modification. Prior to testing the samples, an air background scan was performed, with a minimum of 32 scans to establish a stable baseline spectrum. For sample analysis, a small quantity of either powder or liquid was placed in the light path, and a minimum of 32 scans were recorded for each measurement. The background spectrum obtained from the air scan was then subtracted from the sample spectrum, resulting in the final net spectrum of the sample.

#### 2.4.6. Particle Size Distribution

The particle size distribution of ATH was determined with a BT-2600 laser particle size analyzer from Dandong Baite Instrument Co., Ltd. (Dandong, China).

#### 2.4.7. TGA

The thermogravimetric test was conducted in a nitrogen atmosphere, with a temperature range of 30–1000 °C and a heating rate of 20 °C/min. The equipment used was the NETZSCH TG 209 F3 instrument (NETZSCH Gerätebau GmbH, Görlitz, Bavaria, Germany).

#### 2.4.8. Mechanical Tests

The mechanical properties of the LDPE-based composites were assessed using an ETM 304C universal testing machine from Wance Experimental Equipment Co., Ltd. (Shenzhen, China). The tensile test was conducted with a fixture spacing of 25 mm and a speed of 10 mm/min. For the flexural test, the support gap was 48 mm, and the testing speed was set at 5 mm/min. The Charpy impact test was performed with a test energy of 7.5 J.

## 3. Results and Discussion

### 3.1. Characterization of ATH Particles

#### 3.1.1. Performance Index Test of ATH Particles

**Figure 4:**
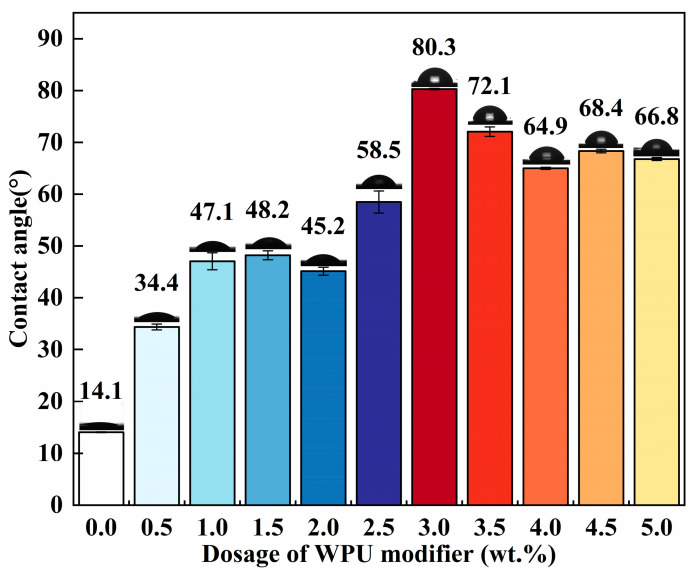
Contact angle of ATH modified with different dosages of WPU, using water as the solvent.

The contact angle results are shown in Figure 4. The unmodified ATH exhibited a wettability contact angle of 14.1°, indicating its hydrophilic nature. The increasing contact angle suggested that the surface of the ATH became more hydrophobic as the dosage of the modifier increased. The contact angle reached a maximum value of 80.3° at a dosage of 3.0 wt.% and was similar to that of dried WPU. This suggests that WPU coated on the surface of the ATH, facilitating their effective incorporation into the LDPE plastic matrix. Upon further increasing the WPU dosage, the contact angle decreased significantly and stabilized between 60° and 70°. This phenomenon can be explained by the fact that excess WPU undergoes self-condensation in water rather than directly coating the ATH surface. The condensed WPU forms multiple layers on the ATH surface. In this multilayer structure, the hydrophilic outer layers of the WPU face outward. This unique structure imparts additional hydrophilicity to the ATH modified with excess WPU, thereby reducing its contact angle. The modification using 3.0 wt.% WPU yielded the best result.

**Figure 5:**
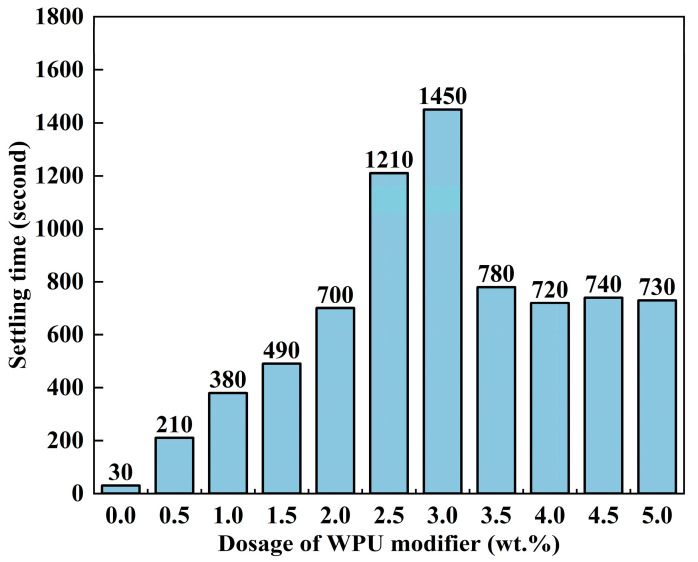
Settling time of ATH with different dosages of modifier.

A key goal of the modification was to enhance the dispersion of ATH powder within the LDPE matrix. Thus, the dispersion and modification effects were assessed by measuring the settling time of ATH powder in kerosene. The results are shown in Figure 5. The modified ATH powder exhibited a much longer settling time compared to the unmodified powder. This suggests that the modification enhances the dispersion of ATH powder in the organic phase. The settling time reached its maximum of 1450 s when the WPU dosage was 3.0 wt.%. Further increases in the modifier content led to a sharp reduction in settling time. This phenomenon occurred because excess modifier causes polyurethane crosslinking on the ATH particles’ surface, leading to aggregation, an increase in particle size and, thus, a decrease in settling time. Based on the combined results of the contact angle and settling time tests, the 3.0 wt.% WPU-modified ATH powder was chosen for further mechanistic studies and the preparation of LDPE-based composites.

#### 3.1.2. Modification Mechanism of ATH

**Figure 6:**
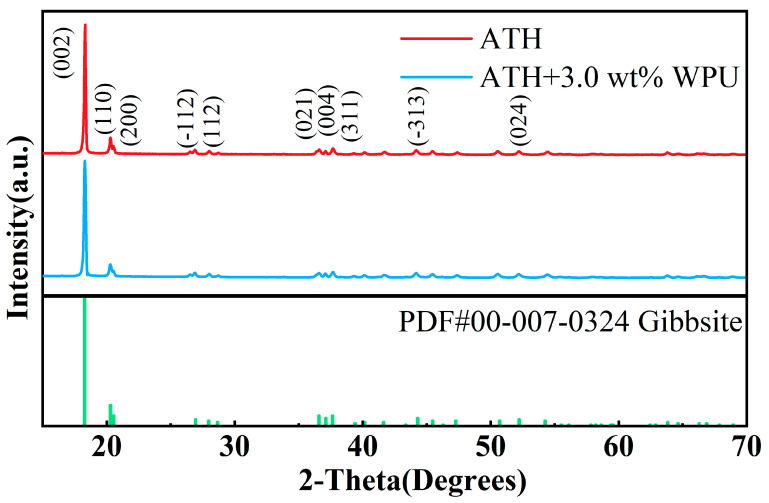
XRD diffraction patterns of ATH, modified ATH, and Powder Diffraction File (PDF) card of gibbsite.

The XRD results are shown in Figure 6. The diffraction peaks at 18.28°, 20.31°, 20.54°, 26.95°, 27.97°, 36.59°, 37.12°, 37.64°, 44.30°, and 52.23° correspond to the (002), (110), (200), (-112), (112), (021), (004), (311), (-313), and (024) crystal planes, respectively. The positions of these diffraction peaks precisely match those recorded in Powder Diffraction File (PDF)#00-007-0324 and other studies of gibbsite [21,22]. There were no significant changes in the diffraction peak positions of ATH before and after modification, indicating that the modification process did not alter the crystal structure. This has been reported in previous studies on similar surface modification treatments [10]. Notably, there was a significant difference in the intensity of the diffraction peaks corresponding to the (002) and (110) crystal planes. The intensity of the diffraction peak for the (002) plane decreased by approximately 15% after modification with 3.0 wt.% WPU. This decrease in peak intensity was due to the surface modification by the organic component, a phenomenon also reported in other surface modification studies of mineral materials [23]. This result further confirms the effectiveness of the modification treatment.

**Figure 7:**
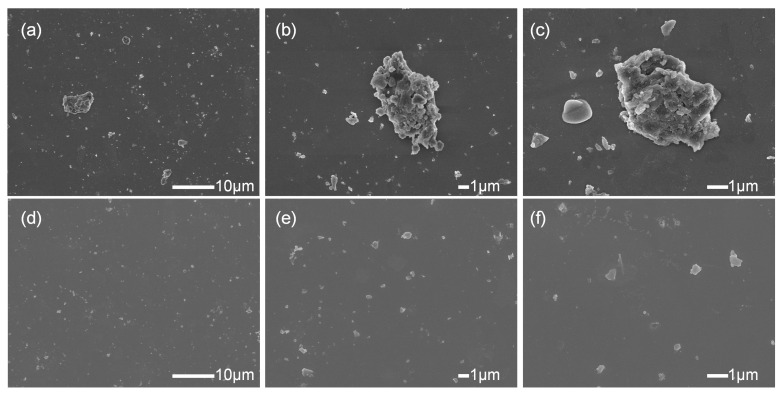
SEM images of (**a**–**c**) ATH and (**d**–**f**) modified ATH.

The SEM images of unmodified and modified ATH powders dispersed in ethanol are shown in Figure 7. It is evident that the monodispersed ATH particles have a particle size of less than 1 μm. The SEM images of unmodified ATH particles clearly show significant aggregation, with most agglomerates ranging from 1 to 10 μm in size. The dispersion of modified ATH particles was significantly improved, with the majority of particles dispersed individually. Even when some particles formed aggregates, these agglomerates were smaller than 1 μm, indicating that the dispersion of ATH powder was enhanced and aggregation was reduced after modification. No coating layer of the WPU modifier was observed on the surfaces of these particles. This observation can be attributed to the breakdown of the WPU modifier under high-energy electron beam bombardment [24,25].

**Figure 8:**
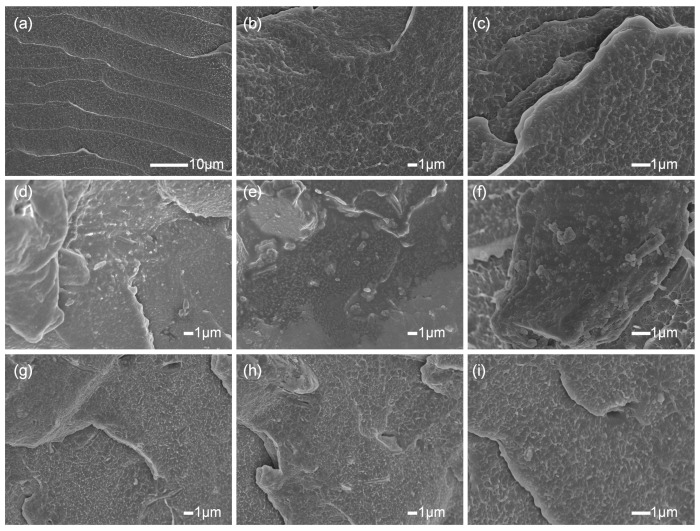
SEM images of (**a**–**c**) LDPE, (**d**–**f**) LDPE/20% ATH, and (**g**–**i**) LDPE/20% ATH + WPU composites.

Figure 8 shows the fracture surface morphology of LDPE-based composites after brittle fracture in liquid nitrogen. The LDPE fracture surfaces shown in Figure 8a–c are relatively smooth, with noticeable “particle-like” or “spherical” aggregates, which are due to the extensive branching and crosslinking of the molecular chains in LDPE. This phenomenon has also been observed in another LDPE-related study [26].

Figure 8d–f show the brittle fracture surfaces of LDPE/20% ATH composites in liquid nitrogen. Bright ATH particles are visible in the images, and their size and morphology are consistent with those shown in Figure 1. The fracture surfaces are no longer perfectly parallel to the observation plane. This indicates the poor compatibility between unmodified ATH particles and the LDPE matrix. This phenomenon can be explained by the fact that, under stress, the weakest interface bonding between ATH and LDPE leads to cracks forming at the interface, which then propagate along the interface. Many of the particles are fully exposed on their surfaces, and the fracture direction is irregular. The dispersion of ATH particles in the LDPE/20% ATH composite is also poor, as observed in Figure 8d–f, with the most prominent aggregation visible in Figure 8f. There are also clear gaps between the aggregated particles, which negatively impact the properties of the LDPE/20% ATH composite.

In contrast, Figure 8g–i show that the dispersion, compatibility, and wettability of modified ATH in LDPE/20% ATH composites are significantly improved. No noticeable gaps or aggregated ATH particles are observed on the fracture surface, and the ATH particles are well dispersed among the LDPE matrix. This improvement is due to the polyurethane coating on the surface of the modified ATH particles, which are chemically bonded, providing strong interface adhesion. The WPU modifier ensures good affinity and wettability of the modified ATH and the LDPE.

**Figure 9:**
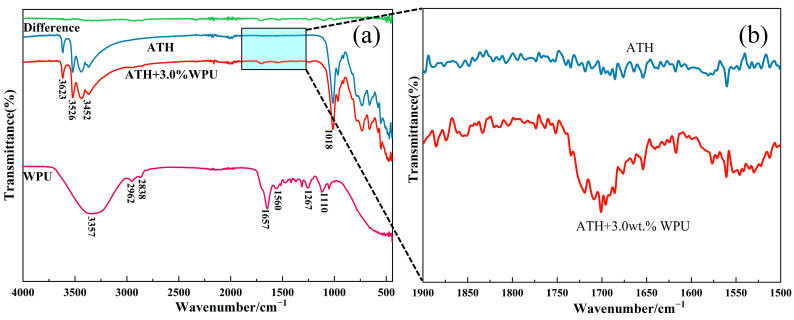
(**a**) FTIR spectra of ATH, modified ATH, WPU, and spectral difference of two kinds of ATH. (**b**) Partial magnification of 1500–1900 cm^−1^ parts.

Figure 9 shows the FTIR spectra of ATH and WPU before and after modification. The absorption bands observed at 3623 cm^−1^, 3526 cm^−1^, and 3452 cm^−1^ in the ATH spectrum correspond to the stretching vibrations of the O-H bond, which are associated with the hydroxyl groups in ATH. This phenomenon of multiple absorption bands around 3500 cm^−1^ has been reported in similar studies on ATH [21,27,28]. The band at 1018 cm^−1^ corresponds to the stretching vibration of the Al-O bond. The absorption bands in the range of 500–600 cm^−1^ are characteristic of the Al-O bending vibration [21,28,29].

In the FTIR spectrum of WPU, the bands at 2962 cm^−1^ and 2838 cm^−1^ are linked to the stretching vibrations of the C-H bonds. The absorption at 3357 cm^−1^ corresponds to the asymmetric stretching vibration of the N-H group, and the in-plane bending vibration of N-H is identified at 1560 cm^−1^. The C=O stretching vibration appears at 1657 cm^−1^, while the C-O stretching vibration is observed at 1110 cm^−1^ [24,25,30].

As shown in the “difference” spectrum in Figure 9, the comparison of the ATH spectra before and after modification reveals that the inclusion of WPU-modified ATH results in the emergence of a new absorption band near 1700 cm^−1^. This band corresponds to the C=O stretching vibration and the in-plane bending vibration of N-H in WPU. This suggests that the modification effectively initiates a condensation reaction between the diisocyanate and polyol groups in WPU and the hydroxyl groups on the ATH surface, leading to the formation of polyurethane chains on the ATH surface.

The XPS spectra of ATH powder before and after modification are shown in Figure S1 in the Supplementary Materials. As shown in Figure S1a,b, the peak intensities of O1s and Al2p in the modified ATH powder are significantly reduced, while the peak intensities of C1s and N1s are notably increased. This is because the modifier grafts onto the ATH surface, introducing additional C-N, C-H, C-C, and C-O bonds. It is important to note that ATH contains a large number of O-H and Al-O bonds, and after modification, a polyurethane coating is formed on the ATH surface, which shields the O-H and Al-O bonds, leading to a decrease in the signal intensity of these bonds.

In Figure S1c, the peaks at 288.71 eV, 286.09 eV, and 284.80 eV are attributed to C-O, C=O bonds, and carbonate, respectively. ATH does not contain any carbon-based chemical bonds, and the appearance of these signals is due to the presence of “dirty carbon” in the testing process and the surrounding air. Such phenomena are commonly observed in the XPS results of other compounds that do not contain carbon elements [1,22,31]. Similarly, in Figure S1d, the binding energy peaks at 289.10 eV, 286.41 eV, 285.51 eV, and 284.80 eV correspond to O-C=O (ester group), C-O, C-N, and C-C/C-H bonds, respectively. It can be seen that, even excluding the signal intensity of dirty carbon, the surface of the modified ATH powder still contains large amounts of C-C/C-H, C-O, and C=O bonds, along with a distinct C-N bond peak. This result indicates that the modification process successfully attached C-N and other carbon-containing chemical bonds to the surface of the ATH, indicating that the modifier successfully coated the ATH surface.

In Figure S1e, the peaks at 531.69 eV and 531.16 eV are attributed to O-H and Al-O bonds, respectively. In Figure S1f, C-O, C=O, H-O, and Al-O bonds correspond to 533.32 eV, 532.35 eV, 531.56 eV, and 531.36 eV, respectively. The peak intensities corresponding to O-H and O-Al bonds in the modified ATH powder are significantly reduced. A similar phenomenon can be observed in the Al2p peaks in Figure 8g,h, where the peak intensity is reduced after modification. This reduction is due to the shielding effect of the long-chain alkyl groups from the polyurethane modifier attached to the ATH surface [23,24,25]. The positions of the Al-OH bonds are 73.95 eV and 73.92 eV [32].

Figure S1i,j show the N1s spectral peaks. The presence of nitrogen on the modified ATH surface leads to the appearance of new peaks in the XPS spectrum. Specifically, the binding energy at 400.01 eV corresponds to the C-N bond, 400.92 eV to N-(COO), and 399.40 eV to N-H. The clear peaks in the XPS results of the modified ATH also confirm that the modifier successfully adhered to the surface of ATH particles, which is consistent with the C-N bond absorption peak observed in the FTIR spectra.

**Figure 10:**
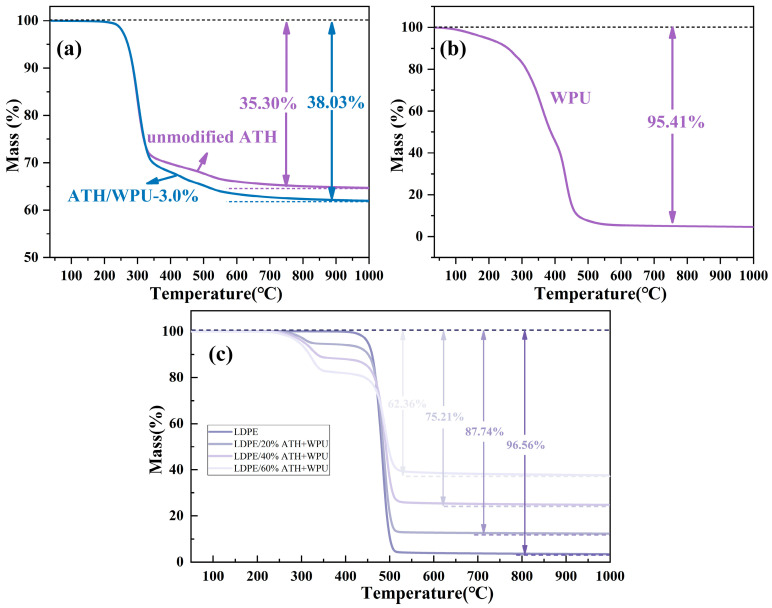
TGA spectra of (**a**) ATH and 3.0wt.% WPU-modified ATH, (**b**) dried WPU, and (**c**) LDPE-based composites.

Figure 10a,b show the thermogravimetric curves of ATH powder before and after modification, as well as dried WPU. The unmodified ATH powder has a mass loss of 35.30 wt.% at 1000 °C, with the main weight loss occurring between 200 °C and 350 °C, caused by the desorption of water and decomposition of the ATH [1,22]. The modified ATH powder shows a mass loss of 38.03 wt.% at 1000 °C, representing an increase of 2.73%, which is consistent with the addition of 3.0 wt.% WPU. The mass loss of the WPU is 95.41 wt.%, with the main weight loss occurring between 300 °C and 500 °C. This temperature range is consistent with the differences in the thermal degradation of both ATH powders, confirming that the WPU modifier added during the modification process was nearly entirely attached to the surface of the ATH powder.

As shown in Figure 10c, the thermal weight loss at 1000 °C for LDPE and LDPE-based composites with 20%, 40%, and 60% ATH contents was 96.56%, 87.74%, 75.21%, and 62.36%, respectively. This thermal weight loss corresponds well with the calculated weight loss based on the thermal weight loss of the modified ATH powder and its content. Around 400 °C, LDPE undergoes minimal thermal decomposition, while ATH decomposes to release water, which evaporates. This is an effective endothermic process, where a large amount of energy is absorbed, and the evaporating water helps dilute flammable pyrolysis gases. The thermal weight loss behavior of LDPE and its composites is consistent with the results from other similar studies [33]. Adding a significant amount of ATH to the LDPE matrix can effectively enhance its flame retardancy [1,7].

### 3.2. Characterization of LDPE-Based Composites

**Figure 11:**
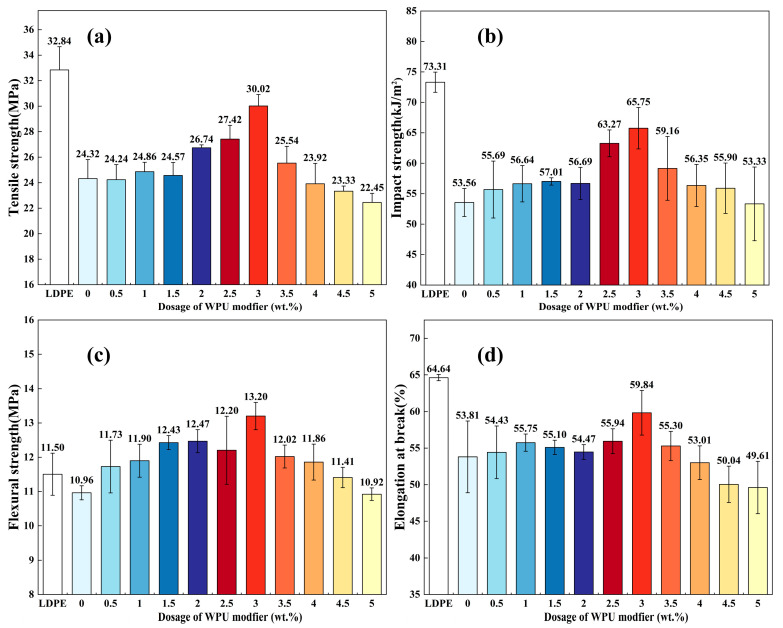
(**a**) Tensile strength, (**b**) flexural strength, (**c**) impact strength, and (**d**) elongation at break of pure LDPE and LDPE/20% ATH composites modified with different dosages of WPU.

Figure 11 shows the mechanical properties of LDPE plastic composites. The tensile strength, impact strength, flexural strength, and elongation at break of pure LDPE plastic splines are 32.84 MPa, 73.31 kJ/m^2^, 11.50 MPa, and 64.64%, respectively. After adding 20% unmodified ATH powder to the LDPE matrix, all mechanical properties of the resulting splines decreased. Specifically, the tensile strength, impact strength, flexural strength, and elongation at break dropped to 23.32 MPa, 53.56 kJ/m^2^, 10.96 MPa, and 53.81%, respectively. This result was due to the poor affinity between ATH powder and the LDPE matrix, as confirmed by the SEM results above. The loose bonding between ATH and LDPE causes voids at the interface, leading to stress concentration at these voids when the strips are subjected to tensile or bending forces. This further extends the cracks and reduces the strength of the strips, resulting in premature fracture. Similarly, during impact testing, the strips absorb less energy due to the poor interface affinity.

The mechanical properties of the LDPE-based composite materials improved gradually with increasing WPU dosage during the modification process. When the WPU dosage reached 3.0 wt.%, the performance of the splines was optimized, with their tensile strength, impact strength, flexural strength, and elongation at break reaching 30.02 MPa, 65.75 kJ/m^2^, 13.20 MPa, and 59.84%, respectively. This improvement in material properties due to WPU modification is significant compared to the composites made with unmodified ATH powder. This enhancement can be attributed to the good wettability, affinity, and dispersion of ATH with the organic phase provided by WPU. In this LDPE-based composite, the ATH powder particles are evenly dispersed and fully coated, enhancing the strength of the splines under bending stress, and allowing the splines to elongate uniformly under tensile stress rather than fracture prematurely due to stress concentration. The results again confirm that the affinity, wettability, and dispersion match the SEM observations. The results of this study are consistent with other findings on the modification of ATH in plastics [1,3,8,9,27]. ATH is widely used as a flame-retardant filler in plastics, and adding a certain amount of ATH to the polymer can enhance the flexural strength. However, this addition typically leads to a reduction in tensile strength and elongation at break [27].

Further increasing the modifier dosage causes a sharp decline in the properties of the composites. This is because an excessive amount of modifier leads to the crosslinking of polyurethane chains on the surface of multiple ATH particles, forming aggregates of particles with sizes reaching tens of micrometers. These aggregates become visible to the naked eye during the preparation of LDPE-based composites and lead to reduced tensile strength, impact strength, flexural strength, and elongation at break.

Table 2 shows the tensile strength, flexural strength, and elongation at break for LDPE-based composite materials with different components.

As shown in Table 2, when 40% and 60% modified ATH powders were added to the LDPE matrix, the composite materials maintained excellent mechanical properties. Due to the reduced proportion of the “softer” LDPE matrix, the tensile and flexural strengths of the composites improved and exceeded the strength of pure LDPE. This result is noteworthy, as it suggests that, in specific application scenarios, adding modified ATH powder as a filler in LDPE can simultaneously provide flame retardancy, high strength, and a significant reduction in preparation costs. Of course, the trade-off is a sharp decrease in the material’s elongation at break. In other similar studies, the tensile strength and elongation at break also decreased with increasing ATH content, and a sharp reduction in elongation at break was observed once a certain loading level was reached [12,34]. However, in this study, the LDPE/ATH composites maintained relatively good flexural and impact strength even at higher ATH loadings. This has not been mentioned in other similar LDPE/ATH composite studies [12,34,35,36].

## 4. Conclusions

In this study, the modification of aluminum hydroxide powder with waterborne polyurethane was successfully carried out, and the effects of the modification on the dispersion, mechanical properties, and thermal stability of ATH in LDPE-based composite materials were thoroughly investigated. The results demonstrate that WPU modification significantly improved the dispersion of ATH in the LDPE, as evidenced by the increased settling time and enhanced surface compatibility. To assess the dispersion, SEM analysis was performed. The SEM images revealed that the dispersion of ATH particles in ethanol and in the prepared LDPE-based composites was significantly more uniform and orderly after modification with the WPU prepolymer. XRD, FTIR, XPS, and SEM analyses confirmed the successful modification of ATH, where WPU formed a coating on the ATH surface and chemically interacted with the hydroxyl groups, leading to improved dispersion and interfacial adhesion.

The modified ATH significantly enhanced the mechanical properties of the LDPE-based composites, achieving a tensile strength of 30.02 MPa, flexural strength of 13.20 MPa, impact strength of 65.75 kJ/m^2^, and elongation at break of 59.84%. These properties reached their peak when 3.0 wt.% WPU was used during the modification process. This highlights the positive role of WPU in improving the strength of the composites, thereby making them more suitable for a wide range of applications. However, excessive WPU content led to the formation of aggregates, which reduced the composites’ mechanical performance.

The modified ATH content in LDPE can be increased to 60%; at this level, the LDPE-based composites exhibited a higher tensile strength of 39.90 MPa and flexural strength of 20.13 MPa compared with the LDPE/20% ATH + WPU composites. The thermogravimetric curves of composites with varying modified ATH content also indicate that large amounts of ATH can decompose before the LDPE plastic. The higher ATH content generates more water during decomposition, and the evaporating water, along with the decomposition reaction, absorbs significant amounts of energy from the system, thereby enhancing the material’s fire resistance. Overall, this study highlights the potential of WPU-modified ATH as an effective flame-retardant and reinforcing filler for LDPE-based composites, offering a promising approach to simultaneously improve material properties and reduce production costs.

## Figures and Tables

**Table 1:** Samples and corresponding descriptions.

Sample	LDPE (wt.%)	ATH (wt.%)	WPU on ATH (%)
LDPE	100	0	0
LDPE/20% ATH	80	20	0
LDPE/20% ATH + WPU	80	20	3.0
LDPE/40% ATH + WPU	60	40	3.0
LDPE/60% ATH + WPU	40	60	3.0

**Table 2:** Tensile strength, elongation at break, and flexural strength of LDPE and LDPE-based composites with different components.

Sample	Tensile Strength (MPa)	Elongation at Break (%)	Flexural Strength (MPa)
LDPE	32.84	63.43	11.50
LDPE/20%ATH	24.32	53.81	10.96
LDPE/20%ATH + WPU	30.02	59.84	13.20
LDPE/40%ATH + WPU	32.87	50.75	13.43
LDPE/60%ATH + WPU	39.90	14.03	20.13

## Data Availability

Data will be made available on request.

## Supplementary Materials

The following supporting information can be downloaded at: https://www.mdpi.com/article/10.3390/polym17050556/s1, Figure S1: XPS spectra of ATH (a, c, e, g, i) and modified ATH (b, d, f, h, j).
