# Supplementary figures and images for: Study on the Mechanical and Thermal Properties of Waterborne Polyurethane-Modified Aluminum Hydroxide and Its Application in LDPE Plastics
Source: Polymers (Basel). 2025 Feb 20;17(5):556. doi: 10.3390/polym17050556 (PMC11902162; doi:10.3390/polym17050556)

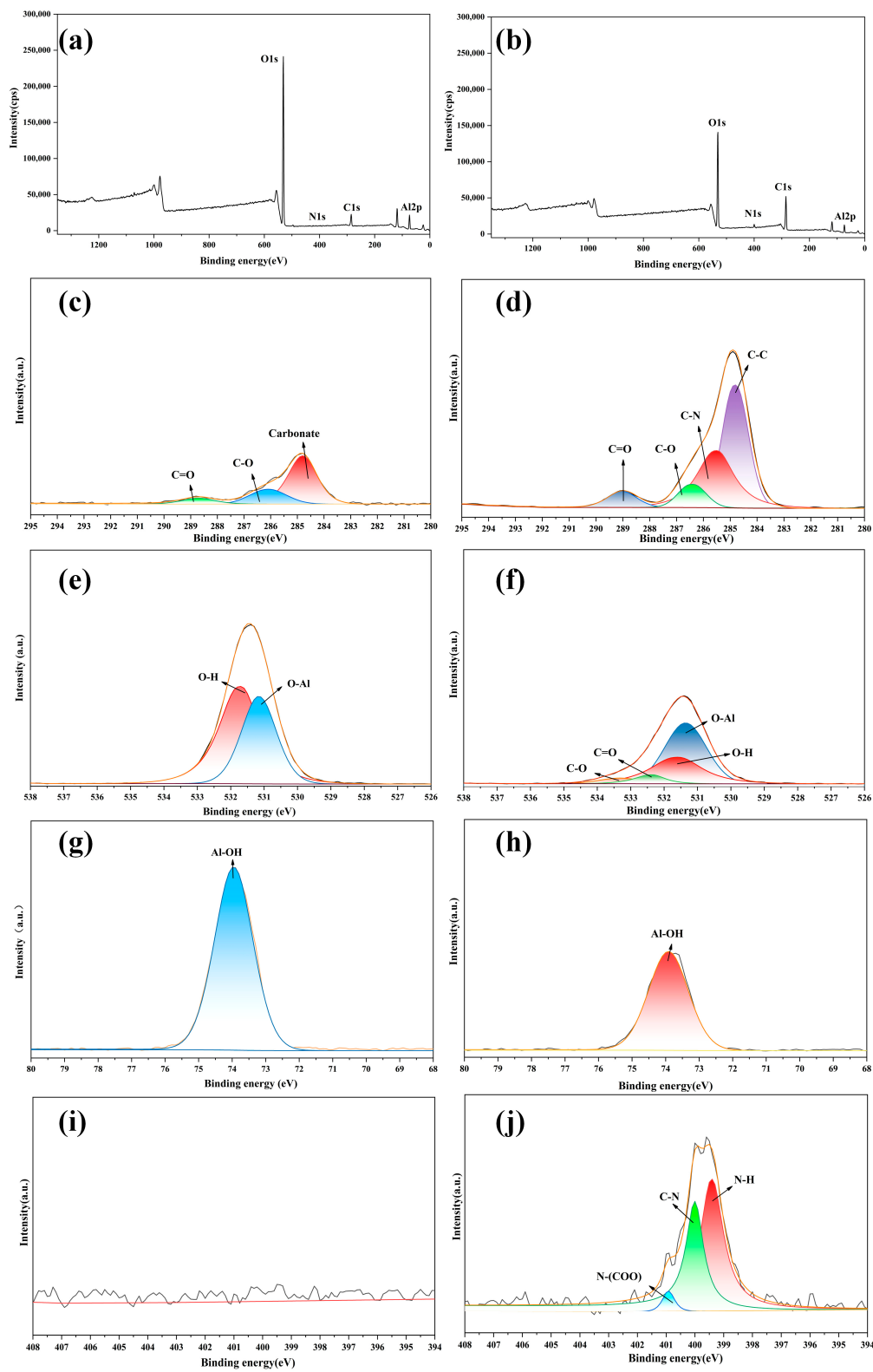

**Figure S1.** XPS spectra of ATH (a, c, e, g, i) and modified ATH (b, d, f, h, j).

Supplement: Supplementary file 1 [file polymers-17-00556-s001.zip › polymers-3469190-supplementary.pdf]
